# Conversations About End-of-Life Decisions in Neonatology: Do Doctors and Parents Implement Shared Decision-Making?

**DOI:** 10.3389/fped.2022.897014

**Published:** 2022-05-23

**Authors:** Esther S. Schouten, Maria F. Beyer, Andreas W. Flemmer, Mirjam A. de Vos, Katja Kuehlmeyer

**Affiliations:** ^1^Division of Neonatology, LMU University Children's Hospital, Dr. v. Hauner, Munich, Germany; ^2^Department of Paediatrics, Emma Children's Hospital, Amsterdam University Medical Centre, Amsterdam, Netherlands; ^3^Institute of Ethics, History and Theory of Medicine, Medical Faculty, LMU Munich, Munich, Germany

**Keywords:** shared decision-making, parents, neonatology, conversations, end-of-life

## Abstract

**Introduction:**

Advances in perinatal medicine have contributed to significantly improved survival of newborns. While some infants die despite extensive medical treatment, a larger proportion dies following medical decision-making (MDM). International guidelines about end-of-life (EOL) MDM for neonates unify in their recommendation for shared decision-making (SDM) between doctors and parents. Yet, we do not know to what extent SDM is realized in neonatal practice.

**Objective:**

We aim at examining to which extent SDM is implemented in the NICU setting.

**Methods:**

By means of Qualitative Content Analysis, audio-recorded conversations between neonatologists and parents were analyzed. We used a framework by de Vos that was used to analyze similar conversations on the PICU.

**Results:**

In total we analyzed 17 conversations with 23 parents of 12 NICU patients. SDM was adopted only to a small extent in neonatal EOL-MDM conversations. The extent of sharing decreased considerably over the stages of SDM. The neonatologists suggested finding a decision together with parents, while at the same time seeking parents' agreement for the intended decision to forgo life-sustaining treatment.

**Conclusions:**

Since SDM was only realized to a small extent in the NICU under study, we propose evaluating how parents in this unit experience the EOL-MDM process and whether they feel their involvement in the process acceptable and beneficial. If parents evaluate their involvement in the current approach beneficial, the need for implementation of SDM to the full extent, as suggested in the guidelines, may need to be critically re-assessed.

## Introduction

Advances in perinatal medicine have contributed to significantly improved survival of newborns. Nevertheless, a substantial number of infants still suffer from terminal or acutely life-threatening health conditions mostly due to (1) extreme preterm birth, (2) congenital malformations or (3) birth related health impairments (4, 5). While some infants die despite extensive medical treatment, a larger proportion dies as a consequence of medical decision-making (MDM) (1, 2). Foregoing life sustaining treatment (LST) may be considered in cases where the patient's prognosis for survival is poor or will most likely be associated with a significantly impaired quality of life (3). Predicting future outcome in such a situation is accompanied by uncertainty (6, 7).

End-of life (EOL)-MDM in neonatology is complex and challenging (7, 8). The medico-ethical principles nonmaleficence, beneficence, respect for autonomy and justice should guide MDM (9). When deciding about health care for children, the “best interest” of the child (10) should be the determining factor (11, 12). Perspectives on what is “best” for a child may differ substantially between parents and medical professionals (6, 13, 14). Differences can occur because of diverging values and preferences that have developed from different cultural backgrounds and experiences (4, 15, 16). It is undoubted that parents should act as surrogate decision-makers for their child in EOL-MDM (11, 17). Yet, the extent of their involvement in the MDM process and the limits of their decision-making authority has been a matter of debate in the last decades (18).

International guidelines about EOL-MDM for neonates are unified in their recommendation for shared-decision making (SDM) between doctors and parents (3, 17, 19–22). Most of them focus on MDM in the case of extreme prematurity at the border of viability. Among others, the German guideline prescribes SDM in such situations (20). It is not always clear, on which concept of SDM these guidelines are based. SDM can be understood as an umbrella term or as a narrow concept (23, 24). As an umbrella term it relates to any form of collaboration between a medical team or specifically a doctor and parents. As a narrow concept it relates to a specific form of partnership between the two parties. Within this partnership, SDM requires a communication process that entails a joint deliberation with an exchange of values and preferences that should be concluded by a decision on which both parties agree upon (25–28). The extent to which doctors and parents join in SDM might vary (29, 30).

So far, the implementation of SDM in practice has been incomplete. De Vos et al. examined the extent of SDM in the pediatric intensive care (PICU) setting (31). They analyzed transcripts of MDM conversations with a pre-developed framework (26, 32). In most cases, there was some extent of sharing information and preferences. Yet, most parents made an effort to participate more actively in the MDM process, while most physicians were carefully preparing the parents for the acceptance of a decision that had been made beforehand by the medical team. A comparable study in the neonatal intensive care unit (NICU) setting is missing. Researchers in neonatology in the UK have analyzed audio-recorded conversations of EOL-MDM conversations. Yet, they analyzed this type of data with other research questions and diverging theoretical frameworks (33–35). In this study, we aim at examining to which extent SDM is implemented in the NICU setting. We conducted a replication of the study design of de Vos et al. to allow for a comparison.

## Materials and Methods

### Study Setting

This study is part of the ENFoLDING (ENd oF Life Decision-making In NeonatoloGy) project funded by the German Ministry of Education and Research (01GY1718). We conducted a prospective study in one of the Level III NICUs of at the LMU medical center, Munich, German. This NICU cares for ~600 neonates per year, of which 1.5% percent die as a result of EOL-MDM (internal data). The current staff leading the EOL-MDM conversations, all senior physicians, were trained in this NICU during their fellowship.

### Data Acquisition

Ethical approval was obtained by the institutional review board at the Medical Faculty of LMU Munich (17–678). Participation was voluntary and could be withdrawn at any time, even after conversations had taken place. All health care personnel was informed about the study goals and gave their written consent to the audio-recording and anonymized transcription of eligible conversations. Participating parents were informed about the study goal, about their participants' rights and written informed consent was obtained prior to the conversations. Before recording of conversations, verbal informed consent of both parties was obtained again.

We adopted consecutive sampling. The inclusion period lasted from May 2018 until October 2020. Eligible participants were identified by the study's PI (ES) or the head of the department (AWF). Potential study participants were approached when their infant was at risk of dying due to a poor health condition. In this publication, we only report cases where conversations took place after the child's birth. We will report our analysis of prenatal conversations elsewhere. All conversations with parents concerning foregoing intensive care treatments were considered eligible.

Conversations were recorded by a portable audio-recorder, uploaded to a personal computer for verbatim transcription by a transcription service following simple transcription rules to Kuckartz and deleted from the recording device (36). Data were handled according to current data protection regulation. This included an anonymization of the data material according to established standards for the anonymization (37). Additional data (underlying diagnosis, survival, withholding/withdrawal LST, duration of medical care, ethnic background of parents, and first language of parents) was obtained from patient charts.

### Data Analysis

We carried out a qualitative content analysis (36). It entails a deductive-inductive-approach to qualitative data analysis. It can be complemented with quantitative analysis (36). It was chosen for the sake of comparability with the study of de Vos et al. It is a useful method to broadly categorize the course of the conversation according to predefined categories. We applied the coding scheme developed by de Vos et al. to the data material (31). Their framework of SDM consists of 3 stages, which may occur consecutively or iteratively (Table 1). Within these stages, they defined codes for specific communicative actions of physicians and parents. We made a minor adaptation to the coding scheme. A code was added to capture when physicians or parents were describing the current situation as a decision-making situation. This code was included, because it marked an important step in the decision-making process. The extent of sharing in the EOL-MDM conversations was evaluated for all stages. The extent of sharing was rated as absent, to a minimal extent, to a moderate extent, to a great extent or to the full extent.

**Table 1:** Three stages of shared decision-making.

**Stage 1 reflects a two-way exchange of information:**
The physician provides information about available treatment options, the benefits and risks, and the potential effects on infants' well-being. The parents provide information about preferences, values, lifestyle, beliefs, and knowledge about the illness, prognosis, and treatment.
**Stage 2 concerns deliberation:**
All parties express and discuss their treatment preferences. This may also include the preference to wait and see or to forgo life sustaining treatment (LST) and redirect care to palliative treatment.
**Stage 3 is about reaching a decision:**
All parties work toward reaching a joint decision to which they all agree.

ES conducted the data analysis. She was trained to apply the coding frame to the conversation transcripts by MdV. Three transcripts were coded by two researchers separately (KK and ESS) and in cases of conflict consensus was sought. The analysis was supported by the computer software MAXQDA (VERBI GmbH, Berlin, Germany).

## Results

### Characteristics of Participants and of the MDM Process

In total, we included 17 conversations with 23 parents of 12 NICU patients. Furthermore, six neonatologists, six nurses and two psychologists participated in the conversations. We included all eligible cases. In four cases, we recorded two meetings of consecutive conversations. The mean duration of the conversations was 24 min (min 11, max 48, std dev. 9). Neonatologists spoke on average 64%, nurses and psychologists spoke 5% and parents 31% of the time. Nurses and psychologists only spoke after being addressed by physicians, e.g., to describe their impression of the infant's condition or to ensure the parents that they were not alone.

Characteristics of the patients and parents are shown in Table 2. Eight patients died after the conversations had taken place and LST was withdrawn. In four cases, clinical deterioration and death was deemed inevitable, for the other four cases prognosis was very poor.

**Table 2:** Characteristics of parents and patients.

**Characteristics**	***N* (%)**
Eligible cases	15 (100%)
Included cases	12 (80%)
Excluded cases*	3 (20%)
**Main diagnosis**	
extreme prematurity	7 (58%)
congenital disorder	3 (25%)
acquired disease or damage	2 (17%)
**Total duration of medical care**	
1-7 days	6 (50%)
1-4 weeks	2 (17%)
>4 weeks	4 (33%)
**Mortality**	
Deceased	8 (67%)
Discharged from NICU	4 (33%)
**Nationality**	
German	10 (83%)
Other**	2 (17%)
**First language parents**	
German	10 (83%)
Other	2 (17%)

*Excluded due to language barriers. **Bosnia, Turkey.

### Communication Behaviors Throughout the MDM Process

Overall, many neonatologists appeared to use a double bind communication in which they suggested to find a decision together with parents, while at the same time seeking parents' agreement to forgo LST. In all cases, the neonatologist started by describing a situation in which a decision on withholding or withdrawing LST might become relevant. This either applied to the current state of the child or to a hypothetical situation in the near future, e.g., due to a possible complication. In nine cases, physicians and parents went through all stages of the SDM process, while in three cases, only stage one or two was being reached within the recorded conversations. Below, we will describe the extent to which these stages were realized.

### Stage 1: Information Exchange

In most recorded conversations, stage 1 was completed (Table 3). Neonatologists typically started the conversations by inviting parents to share their understanding of the current situation. Almost all parents provided information regarding their view on the current condition of their child. After acknowledging the parents' answers, an update on the current medical situation was provided. This included the current deterioration of the child's condition or complications accompanying the current treatment. In all conversations, neonatologists explained the patient's short- and/or long-term prognosis. None of the parents were ever asked to present their view on possible treatment options or to share their opinion on prognosis. One third of the parents explicitly asked doctors about the prognosis. Although none of the parents were asked to do so, one third spontaneously shared their opinion on their child's prognosis. In two thirds of the conversations, neonatologists provided their opinion on remaining treatment options including their benefits and risks. Parents asked questions to deepen their understanding of their child's medical problems. Neonatologists frequently referred to drawings or ultrasound or x-ray pictures to illustrate health problems or complications such as intraventricular hemorrhage or pneumothorax. Parents frequently asked for reasons for the complications which had occurred or why treatment so far was not successful.

**Table 3:** Stage 1: exchange of information.

**Coded behaviors**	**From neonatologist to parents**		**From parents to neonatologist**	
	**Cases (*n* = 12) N (%)**	**Illustrative quotes**	**Cases (*n* = 12) *N* (%)**	**Illustrative quotes**
1. Asking for information about actual situation and treatment effects	11 (92%)	“You are here every day, you kanguru with him. What is your impression? How has he been doing the past few days?” (#10)	8 (67%)	“But his heart is still good right?” (#11)
2. Providing information about actual situation and treatments effects	12 (100%)	“The main problem is that the kidneys do not function, not a drop of urine has come. Two days ago, we hoped for recovery but until now we haven't seen any effect of the treatment.” (#4)	11 (92%)	“It is hard to just sit there and wait: she does not move, she does not look at us.” (#5)
3. Asking for information about prognosis	0 (0%)	–	5 (42%)	“What does that mean: ‘when things do not get better'? Does it mean we might have a disabled child?” (#1)
4. Providing information on prognosis	12 (100%)	“The perforation of the bowel will lead to an infection of the abdomen with peritonitis, by then your daughter will be severely affected.” (#2)	4 (33%)	“She has a bleeding in her brain, not a small bleeding but a severe bleeding and there will probably be long term damage to her brain.” (#1)
5. Asking for information about remaining options, including pros and cons	0 (0%)	–	5 (42%)	“How can you treat this intracranial bleeding? Is there something you can do?” (#3)
6. Providing information about remaining options, including pros and cons	8 (67%)	“There are two options: we can either continue the cooling therapy for 72 h and see how she is doing afterwards or we can redirect care because we think this life sustaining therapy is not leading anywhere.” (#8)	2 (17%)	“I was wondering, can't we leave her in peace for a day? Just give her a rest and not performing any x-ray today?” (#3)
7. Asking for a summary	3 (25%)	“Let's start of by checking whether we are all on the same page, can you tell me what you've understood so far?” (#7)	0 (0%)	—–
8. Providing a summary	1 (8%)	“Before we talk about the operation, let me summarize the current situation.” (#12)	1 (8%)	“Okay, back to Max, I would really like to tell you what I understood so far.” (#5)
9. Inviting to ask additional questions	2 (17%)	“Is there something you did not understand, something that is not clear to you?” (#7)	0 (0%)	—–

### Stage 2: Joint Deliberation

Whereas in many conversations some kind of deliberation took place, other actions typical for this stage were largely missing (Table 4.). In all cases, the neonatologists expressed a decision situation. They either emphasized the option of foregoing LST in the current situation or referred to a possible future scenario. In the latter case, they described that a further deterioration of the child's condition could require a deliberation “how much intensive care is still sensible” (case #1, case #2, case #7). None of the parents were asked which role they preferred in EOL-MDM. In one case, it was explicitly suggested that in such situations the medical team decides together with the patient's family.

**Table 4:** Stage 2: deliberation.

**Coded behaviors**	**From neonatologist to parents**		**From parents to neonatologist**	
	**Cases (*n* = 12) *N* (%)**	**Illustrative quotes**	**Cases (*n* = 12) *N* (%)**	**Illustrative quotes**
0. Situation defined as decision-making situation	12 (100%)	“Hopefully, the situation will not progress, because if it gets worse and Katie's situation deteriorates, we need to discuss how much intensive care is sensible.” (#1)	0 (0%)	—–
1. Asking which role in decision-making would be preferred	0 (0%)	—–	0 (0%)	—–
2. Providing information about which role in decision-making would be preferred	1 (8%)	“Well, in most cases, we decide together with the family.” (#11)	0 (0%)	—–
3. Asking about values and preferences regarding continuation or discontinuation	9 (75%)	“This is the moment, one needs to consider, we together, need to consider what our hopes and goals for Paula are, what do you wish for Paula?” (#8)	0 (0%)	—–
4. Providing information about values and preferences regarding continuation or discontinuation	10 (83%)	“We could keep him alive on the respirator, but I do not think that is the right thing for Mohammed.” (#10)	7 (58%)	“In my opinion, we have to give it a try. To be honest, well of course, I'm not a doctor, but to say now; we won't do anything anymore, I cannot find it in my heart.” (#4)
5. Expressing objections to the other's preferences	0 (0%)	—–	0 (0%)	—–
6. Inviting to share emotions	1 (8%)	“What do you worry about the most?” (#4)	0 (0%)	—–
7. Expressing emotions of grief, fear, despair, and frustration	0 (0%)	—–	7 (58%)	“[crying] well, we'll probably have to let her go, let her go in peace.” (#1)
8. Acknowledging emotions	1 (8%)	“I know, this insecurity is frightful.” (#8)	0 (0%)	—–

Sharing preferences and values between parents and physicians took place in the majority of cases. Yet, the extent of sharing varied greatly between cases. The majority of the neonatologists (in nine out of twelve cases) invited the parents to express their preferences and values concerning continuation or discontinuation of LST. Neonatologists did so by describing several options and subsequently stating that in medicine not everything possible needs to be done. Values and preferences voiced by parents were seldomly challenged by the neonatologists. Neonatologists rarely reacted to parents' responses, nor did they inquire after the background of these wishes. In most cases, the neonatologist suggested a discontinuation of LST. At that point, they were speaking of “we,” thereby referring to the medical team. This preferred option was verbalized as when the clinical situation of the child deteriorated quickly, the child should be “allowed to go” (case #1) or “we should allow nature to take its course” (case #7). In cases of anticipated deterioration, the burden of LST was addressed as an import aspect to consider in MDM. The majority of parents (10 of 12 cases) communicated a preference. In all but one cases, their preferences were in alignment with the suggestion. Only in one case, a conflict occurred. These parents voiced that they “could not find it in their heart” not to try one more treatment option, although they did not want to continue intensive care “at all costs” (case #4). As the clinical situation of their baby did not improve the following day, the parents agreed with the withdrawal of LST. In two cases, parents expressed wanting to protect their child from a life that in their eyes seemed not worth living. Parents articulated their worries about their child's suffering. Parents frequently reacted emotionally when it became clear that a decision had to be made. They were especially emotional when they stated their agreement with a discontinuation of LST. Parents often justified their agreement with the wish to alleviate the suffering of their child.

### Stage 3: Reaching a Consented Decision

Only a subset of the recorded conversations entailed a decision (9 of 12 cases). In the conversations which referred to stage 3, neonatologists mostly sought parents' agreement with a proposed decision (Table 5). In two cases, the neonatologist asked the parents when and which decision should be made. In one case, this question came from the parents. In half of the cases, the neonatologist informed the parents about the kind of decision that the medical team had made. In three cases the decision was primarily verbalized by the parents. This included two cases of severely asphyxiated infants, in which the parents had received information from the pediatric neurologist about the poor neurological prognosis of their child before the conversation with the neonatologist. Both couples suggested forgoing LST because of the expected poor quality of life of their child. They stated this was not “the life their child would have wanted” (case #6 and #8). In the remaining case, the parents wished to continue LST for now and give dialysis a try, thereby opposing the medical team's suggestion.

**Table 5:** Stage 3: reaching a decision.

**Coded behaviors**	**From neonatologist to parents**		**From parents to neonatologist**	
	**Cases (*n* = 12) *N* (%)**	**Illustrative quotes**	**Cases (*n* = 12) *N* (%)**	**Illustrative quotes**
1. Asking whether and which decision should be made	2 (17%)	“What would you prefer? We could give it a try and remove him from the respirator. If he has trouble breathing, we can support him with non-invasive oxygen but we need to know whether or not you want us to intubate him again in case he will not be able to breath on his own sufficiently.” (#10)	1 (8%)	“If nothing changes until this afternoon, is this the moment we have to decide?” (#8)
2. Informing about decision being reached	6 (50%)	“As we discussed yesterday; we'll give him a chance. But when it's not working out or new complications arise, we need to let him go.” (#4)	3 (25%)	“Nature took its course and we decided, for ourselves, we believe strongly she is a very special child, and dear god needs her now. We have to let this new guardian angel go to heaven now, that's what we decided.” (#8)
3. Stressing that team should make final decision	2 (17%)	“You do not have to decide. You cannot decide, that would be unbearable for a parent, to decide: do we continue or not.” (#4)	1 (8%)	“[Father translates for his wife] She says she can't decide. We depend on your expertise, you have been here before and know what is best.” (#9)
4. Asking for agreement regarding proposed decision	6 (50%)	“Well, this means, we would not initiate resuscitation, we would say that's it, it is fine, we've tried, we really did everything we could, maybe even a bit more than that'. Are you ok with that decision?” (#11)	0 (0%)	—–
5. Expressing agreement	3 (25%)	“I think, we can go along with that and give the dialysis a try, if that is what you really want.” (#4)	6 (50%)	“[crying] yes, let's do it like that, there is no way around.“ (#2)
6. Expressing dissent	0 (0%)	—–	0 (0%)	—–
7. Expressing worries about suffering	0 (0%)	—–	6 (50%)	“[crying] surely he is not in pain?” (#5)

In the other three out of twelve cases, no explicit decision to forgo LST was reached. Either the conversation was used to prepare parents for an anticipated deterioration of the child's condition (two cases), or the parents expressed their thoughts on giving their son the opportunity to “go in peace” (case #5). The medical team adopted a “wait and see” approach. Christening was arranged. The baby deteriorated very fast after the christening and died before parents and the neonatologist could meet again.

In two thirds of the conversations that concluded with a decision (six out of nine cases), the neonatologist asked the parents for their agreement on the suggested option. In three of these cases, the neonatologist suggested to withdraw LST on the basis of futility arguments. The parents agreed and acknowledged that everything possible had been done and that their child's death was inevitable. In the other three cases, the neonatologist suggested to continue LST for now, but to withhold resuscitation if the child had a cardiac arrest. In none of these cases there was an open conflict in which disagreement with another person's position was being expressed. In one case, parents did at first not agree with withdrawing LST and wished for another treatment option. The neonatologist suggested a trial, but also proposed to forgo LST if the child's condition did not improve.

In many conversations, parents expressed their worries that their child would suffer and experience pain. All neonatologists ensured parents that it was their highest priority to make their child feel as comfortable as possible, especially after withdrawing LST.

### The Extent of Sharing in the Decision-Making

In Table 6 an overview of the extent of sharing is depicted. In all stages of the decision-making process, there was some sharing of information between neonatologists and parents. Yet, the extent of sharing varied throughout the different stages of the decision-making process and between cases (Table 6). The extent of sharing was especially large in the first phase of MDM in which neonatologists explained the infant's condition and prognosis. It was rather small in the following phases of MDM.

**Table 6:** Extent of sharing.

**Process of decision-making**	**Characteristics of shared process**	**Extent of sharing identified**
		**(absent, minimal, moderate, great, full)**
Stage 1: Providing	1. Exchange of information	
and receiving Information	1.1 Neonatologist informs parents about actual situation, prognosis,	full
	treatment options, and their risk and benefits	
	1.2 Parents inform neonatologist about their observations and considerations	great
	2. Helping parents understand	
	2.1 By inviting to ask questions	minimal
	2.2 By checking understanding	moderate
Stage 2:	3. Discussing which role parents prefer to have in decision-making	absent
Deliberating	4. Discussing treatment preferences	
	4.1. Neonatologist expresses preference.	great
	4.2. Parents express preference.	great
	4.3. Exchanging underlying values and deliberations	minimal
Stage 3: Reaching	5. Making the final decision together.	minimal
a Decision	6. Reaching agreement (eventually) about the most appropriate decision	full

*Adapted from de Vos et al. (31)*.

*Absent, no sharing; minimal extent, behavior basically registered; moderate extent, behavior globally registered; great extent, behavior in detail registered; full extent, behavior exhaustingly registered*.

## Discussion

International guidelines underline that neonatologists should adopt an approach of SDM when deciding about foregoing LST (3, 17, 19, 20, 22). This requires a partnership with parents. Within this partnership, SDM describes a communication process that entails a mutual exchange of information, values and preferences and that should result in a joint decision to which both parties agree (26, 27). To our knowledge, this is the first study that aimed at evaluating to what extent SDM is put into practice in the NICU. It is also the first German study analyzing natural EOL-MDM conversations between neonatologists and parents. Our study shows that in a German unit, SDM was adopted but only to a small extent in neonatal EOL-MDM conversations. The extent of sharing decreased considerably over the stages of SDM and among cases. Overall, many neonatologists appeared to use a double bind communication in which they suggested to find a decision together with parents, while at the same time seeking parents' agreement to forgo LST.

The EOL-MDM conversations largely evolved around the exchange of information about the infant's current condition and prognosis. The deliberation process, which is characterized in the framework by each party providing and challenging values and preferences concerning forgoing LST, only took place to a minimal extent in the conversations. This pattern is similar to the pattern in the study of de Vos et al. in the PICU (31). While in the Dutch PICUs most parents made an effort to actively participate in EOL-MDM, in our study parents showed less efforts to participate more in the process. When parents provided their own preferences and values, they frequently did so after actively being prompted by the neonatologist. Moreover, neonatologists acknowledged parents' preferences, but seldomly challenged them. A true dialogue evolved only sporadically. Although the minimal requirements for deliberation were fulfilled, there was rarely an active discussion to reach a bilateral understanding of each other's values and preferences, an important aspect of a shared approach (38).

Recent studies show that the extent to which doctors and parents prefer to collaborate varies (24, 30). The complexity of the medical situation and the substantial chance of an inevitable outcome may cause neonatologists to strongly feel their decisional responsibility (39, 40). A survey among Belgian neonatologists reported that doctors claim to be responsible for the final decision, not the parents (41). The neonatologists in this study frequently acted in line with this perspectives and sometimes even explicitly mentioned it in the conversations.

An important argument for the recommendation of SDM as the preferred decision-making strategy in EOL-MDM is that parents want to be involved in the MDM process. The variation in the extent of sharing may also be caused by different parents having different expectations and wishes concerning their role in the EOL-MDM. Some parents clearly want to know all the details and share in the responsibility, while others specifically state that they have all the faith in the medical team and think the medical team knows best (13, 16, 23, 42).

The framework adopted in this study is not the only conceptualization of SDM. Researchers in neonatology have proposed a myriad of SDM frameworks. The approach described by Caeymaex et al. entails a discussion on the nature of the decision, with exchange of relevant medical information (including medically reasonable alternatives), followed by the exchange of family values and preferences and leading to a parental choice about the most appropriate decision. In our NICU, the EOL-MDM approach did not lead to a parental choice. A more tailored approach to SDM is also recommended in the literature (40, 43). This approach is described by Janvier et al. for EOL-MDM in the NICU. The use of the mnemonic SOBPIE (situations, opinion and options, basic human interactions, parents' personal experiences/stories/concerns/goals, information, emotions) is suggested to personalize conversations with parents and help them arrive at satisfying decisions (43). This includes that parents should be asked which role they want to adopt in the SDM process. In our study, this approach was never adopted. Perhaps, neonatologists were reluctant to openly deliberate with parents on their decision-making role because they were afraid it would make parents feel responsible for making the final decision. Decisional regret and guilt have been described as negative consequences of participating in EOL-MDM (30, 40, 42). Furthermore, all parents facing EOL-decisions in the NICU are in a vulnerable emotional position beforehand; many of them becoming parents for the first time and all mothers being in an exceptional physical and emotional condition as they just gave birth. Hendriks and colleagues describe an approach which they call the “ethical model” of decision-making. This decision-making strategy entails that the medical team provides parents with comprehensive medical information to enable them to understand the best treatment option and give them the opportunity to ask clarifying questions. Next, the medical team voices a treatment recommendation and asks the parents for their consent. This treatment recommendation is based on the medical facts, the infant's best interests and the parents' values which are determined indirectly (44). This approach shows similarities with the approach that neonatologists chose in our study.

### Limitations

Our study has several limitations. The recorded MDM conversations only took place in one NICU. Communication strategies may have been acquired through model learning, providing a unit-specific strategy of communicating with parents. Our findings can therefore not be extrapolated to all German NICUs.

Furthermore, the recorded conversations are only extracts of a complex and multifaceted MDM process. Only formal conversations with parents and neonatologists were recorded, leaving out the informal encounters at the bedside or the conversations with neurologists or the nursing staff before and after these official meetings. Furthermore, team meetings were not recorded. We might have missed out on crucial activities of deliberation and conflict resolution.

The predefined framework we used in our study did not fully fit the natural course of the conversations. With the coding grid, we could show whether preferences and values were voiced by both parties. Yet, it was not specific enough to show whether a joint deliberation of values and preferences had occurred and how it had occurred. The “ethical model” described by Hendriks et al. could be a more suitable framework to describe EOL-MDM in the NICU under study (45). Yet, it has been criticized as explicitly not sufficient to speak of SDM (46). To extend the investigation of actual communication practices to broader research questions, such as the social interaction during SDM and the precise communicative implementation of SDM, we think that a mixed methods design would be a more suitable choice in which video-recordings of a broad range of natural conversations are inductively analyzed and compared with the outcomes of retrospective interviews.

## Conclusion

The EOL-MDM process in the recorded conversations in this German NICU could be, using the framework of de Vos et al., identified as SDM. However, it was only realized to a small extent. Specific research with the neonatal staff could provide insights to understand why the approach in this NICU differs from the recommendations. Furthermore, interviews with affected parents in this unit could be of great interest. It could evaluate how parents in this unit have experienced the EOL-MDM process and whether they feel their involvement in the process was acceptable and beneficial. If parents are satisfied with their involvement and consider it appropriate, then it could be argued that SDM is not the only suitable approach to a collaborative EOLD-MDM. Different parents may prefer different roles in EOL-MDM, and the need for implementation of SDM to the full extent as suggested in the guidelines may need to be critically re-assessed.

## Data Availability Statement

The datasets presented in this article are not readily available because the data is very sensitive: transcripts of parent-physician conversations. Requests to access the datasets should be directed to esther.schouten@med.uni-muenchen.de.

## Ethics Statement

Ethical approval was obtained by the Institutional Review Board at the Faculty of LMU Munich (17–678). The patients/participants provided their written informed consent to participate in this study.

## Author Contributions

ES had primary responsibility for protocol development, patient screening, enrollment, outcome assessment, preliminary data analysis, and writing the manuscript. MB was responsible for enrollment, outcome assessment, and contributed to the writing of the manuscript. AF was responsible for patient screening and contributed to the writing of the manuscript. MV supervised the design and contributed to the writing of the manuscript. KK participated in the data analysis, contributed to the analytical framework and to the writing of the manuscript. All authors contributed to the article and approved the submitted version.

## Funding

ES, MB, and KK were supported by a young researcher grant by the German Ministry of Education and Research (Bundesministerium für Bildung und Forschung) (Grant no. 01GY1718).

## Conflict of Interest

The authors declare that the research was conducted in the absence of any commercial or financial relationships that could be construed as a potential conflict of interest.

## Publisher's Note

All claims expressed in this article are solely those of the authors and do not necessarily represent those of their affiliated organizations, or those of the publisher, the editors and the reviewers. Any product that may be evaluated in this article, or claim that may be made by its manufacturer, is not guaranteed or endorsed by the publisher.

## References

[1] Trowbridge A Walter JK McConathey E Morrison W Feudtner C. Modes of death within a children's hospital. Pediatrics. (2018) 142:e20174182. 10.1542/peds.2017-4182 30232217

[2] Dupont-Thibodeau A Langevin R Janvier A. Later rather than sooner: the impact of clinical management on timing and modes of death in the last decade. Acta Paediatr. (2014) 103:1148-52. 10.1111/apa.12747 25040044

[3] American American Academy of Pediatrics Committee on F Newborn Bell EF. Noninitiation or withdrawal of intensive care for high-risk newborns. Pediatrics. (2007) 119:401-3. 10.1542/peds.2006-3180 17272630

[4] Verhagen AA Janvier A Leuthner SR Andrews B Lagatta J Bos AF. Categorizing neonatal deaths: a cross-cultural study in the United States, Canada, and the Netherlands. J Pediatr. (2010) 156:33-7. 10.1016/j.jpeds.2009.07.019 19772968

[5] Weiner J Sharma J Lantos J Kilbride H. How infants die in the neonatal intensive care unit: trends from 1999 through 2008. Arch Pediatr Adolesc Med. (2011) 165:630-4. 10.1001/archpediatrics.2011.102 21727274

[6] LoRe D Mattson C Feltman DM Fry JT Brennan KG Arnolds M. Physician perceptions on quality of life and resuscitation preferences for extremely early newborns. Am J Perinatol. (2021). 10.1055/s-0041-1733782. [Epub ahead of print]. 34352923

[7] Wood K Di Stefano LM Mactier H Bates SE Wilkinson D. Individualised decision making: interpretation of risk for extremely preterm infants-a survey of UK neonatal professionals. Arch Dis Child Fetal Neonatal Ed. (2021). 3:281–8. 10.1136/archdischild-2021-322147 34413095 PMC9046748

[8] Prentice TM Gillam L Davis PG Janvier A. The use and misuse of moral distress in neonatology. Semin Fetal Neonatal Med. (2018) 23:39-43. 10.1016/j.siny.2017.09.007 28964686

[9] Beauchamp TL: CJF. Principles of Biomedical Ethics. 8th ed. New York, NY: Oxford University Press (2019).

[10] Patel RM. Short- and long-term outcomes for extremely preterm infants. Am J Perinatol. (2016) 33:318-28. 10.1055/s-0035-1571202 26799967 PMC4760862

[11] Ladd RE Mercurio MR. Deciding for neonates: whose authority, whose interests? Semin Perinatol. (2003) 27:488-94. 10.1053/j.semperi.2003.10.008 14740947

[12] Stokes TA Kukora SK Boss RD. Caring for families at the limits of viability: the education of Dr Green. Clin Perinatol. (2017) 44:447-59. 10.1016/j.clp.2017.01.007 28477671

[13] Tucker Edmonds B Hoffman SM Laitano T Bhamidipalli SS Jeffries E Fadel W. Values clarification: eliciting the values that inform and influence parents' treatment decisions for periviable birth. Paediatr Perinat Epidemiol. (2020) 34:556-64. 10.1111/ppe.12590 31637742

[14] Saigal S Stoskopf BL Feeny D Furlong W Burrows E Rosenbaum PL. Differences in preferences for neonatal outcomes among health care professionals, parents, and adolescents. JAMA. (1999) 281:1991-7. 10.1001/jama.281.21.1991 10359387

[15] Boland RA Cheong JLY Stewart MJ Kane SC Doyle LW. Disparities between perceived and true outcomes of infants born at 23-25 weeks' gestation. Aust N Z J Obstet Gynaecol. (2021). 10.1111/ajo.13443. [Epub ahead of print]. 34687048

[16] Sharman M Meert KL Sarnaik AP. What influences parents' decisions to limit or withdraw life support? Pediatr Crit Care Med. (2005) 6:513-8. 10.1097/01.PCC.0000170616.28175.D9 16148808

[17] Biotethics NCo. Critical Care Decisions in Fetal and Neonatal Medicine: Ethical Issues. London: Biotethics NCo (2006).

[18] Sullivan A Cummings C. Historical perspectives: shared decision making in the nicu. Neoreviews. (2020) 21:e217-25. 10.1542/neo.21-4-e217 32238484 PMC8049458

[19] Mactier H Bates SE Johnston T Lee-Davey C Marlow N Mulley K. Perinatal management of extreme preterm birth before 27 weeks of gestation: a framework for practice. Arch Dis Child Fetal Neonatal Ed. (2020) 105:232-9. 10.1136/archdischild-2019-318402 31980443

[20] Bührer C Felderhoff-Muser U Gembruch U Hecher K Kainer F Kehl S. Frühgeborene an der Grenze der Lebensfähigkeit (Entwicklungsstufe S2k, AWMF-Leitlinien-Register Nr. 024/019, Juni 2020). Z Geburtshilfe Neonatol. (2020) 224:244–54. 10.1055/a-1230-0810 33075837

[21] Lemyre B Moore G. Counselling and management for anticipated extremely preterm birth. Paediatr Child Health. (2017) 22:334-41. 10.1093/pch/pxx058 29485138 PMC5804811

[22] Dageville C Betremieux P Gold F Simeoni U Working Working Group on Ethical Issues in P. The French Society of Neonatology's Proposals for neonatal end-of-life decision-making. Neonatology. (2011) 100:206-14. 10.1159/000324119 21471705

[23] Brinchmann BS Forde R Nortvedt P. What matters to the parents? A qualitative study of parents' experiences with life-and-death decisions concerning their premature infants. Nurs Ethics. (2002) 9:388-404. 10.1191/0969733002ne523oa 12219402

[24] Caeymaex L Jousselme C Vasilescu C Danan C Falissard B Bourrat MM. Perceived role in end-of-life decision making in the nicu affects long-term parental grief response. Arch Dis Child Fetal Neonatal Ed. (2013) 98:F26-31. 10.1136/archdischild-2011-301548 22732115

[25] Charles C Gafni A Whelan T. Shared decision-making in the medical encounter: what does it mean? (or it takes at least two to tango). Soc Sci Med. (1997) 44:681-92. 10.1016/S0277-9536(96)00221-3 9032835

[26] Charles C Gafni A Whelan T. Decision-making in the physician-patient encounter: revisiting the shared treatment decision-making model. Soc Sci Med. (1999) 49:651-61. 10.1016/S0277-9536(99)00145-8 10452420

[27] Elwyn G Frosch D Thomson R Joseph-Williams N Lloyd A Kinnersley P. Shared decision making: a model for clinical practice. J Gen Intern Med. (2012) 27:1361-7. 10.1007/s11606-012-2077-6 22618581 PMC3445676

[28] Opel DJ. A 4-step framework for shared decision-making in pediatrics. Pediatrics. (2018) 142(Suppl 3):S149-56. 10.1542/peds.2018-0516E 30385621

[29] Caeymaex L Speranza M Vasilescu C Danan C Bourrat MM Garel M. Living with a crucial decision: a qualitative study of parental narratives three years after the loss of their newborn in the nicu. PLoS ONE. (2011) 6:e28633. 10.1371/journal.pone.0028633 22194873 PMC3237456

[30] Baughcum AE Fortney CA Winning AM Shultz EL Keim MC Humphrey LM. Perspectives from bereaved parents on improving end of life care in the nicu. Clin Pract Pediatr Psychol. (2017) 5:392-403. 10.1037/cpp0000221

[31] de Vos MA Bos AP Plotz FB van Heerde M de Graaff BM Tates K. Talking with parents about end-of-life decisions for their children. Pediatrics. (2015) 135:e465-76. 10.1542/peds.2014-1903 25560442

[32] White DB Braddock CH 3rd Bereknyei S Curtis JR. Toward shared decision making at the end of life in intensive care units: opportunities for improvement. Arch Intern Med. (2007) 167:461-7. 10.1001/archinte.167.5.461 17353493

[33] Shaw C Connabeer K Drew P Gallagher K Aladangady N Marlow N. Initiating end-of-life decisions with parents of infants receiving neonatal intensive care. Patient Educ Couns. (2020) 103:1351-7. 10.1016/j.pec.2020.02.013 32111382

[34] Marlow N Shaw C Connabeer K Aladangady N Gallagher K Drew P. End-of-life decisions in neonatal care: a conversation analytical study. Arch Dis Child Fetal Neonatal Ed. (2021) 106:184-8. 10.1136/archdischild-2020-319544 32943530

[35] Shaw C Stokoe E Gallagher K Aladangady N Marlow N. Parental involvement in neonatal critical care decision-making. Soc Health Illn. (2016) 38:1217-42. 10.1111/1467-9566.12455 27666147

[36] Kuckartz U. Qualitative Inhaltsanalyse : Methoden, Praxis, Computerunterstützung. 4th ed. Weinheim Basel: Juventa Verlag ein Imprint der Julius Beltz GmbH & Company KG (2018).

[37] Meyermann A Porzelt M. Hinweise Zur Anonymisierung Von Qualitativen Daten. In: Forschungsdaten Bildung Informiert (2014) p. 6–12.

[38] Stiggelbout AM Pieterse AH De Haes JC. Shared decision making: concepts, evidence, and practice. Patient Educ Couns. (2015) 98:1172-9. 10.1016/j.pec.2015.06.022 26215573

[39] Richards CA Starks H O'Connor MR Bourget E Hays RM Doorenbos AZ. Physicians perceptions of shared decision-making in neonatal and pediatric critical care. Am J Hosp Palliat Care. (2018) 35:669-76. 10.1177/1049909117734843 28990396 PMC5673589

[40] Parish O Williams D Odd D Joseph-Williams N. Barriers and facilitators to shared decision-making in neonatal medicine: a systematic review and thematic synthesis of parental perceptions. Patient Educ Couns. (2021). 10.1016/j.pec.2021.08.033. [Epub ahead of print]. 34503868

[41] Aujoulat I Henrard S Charon A Johansson AB Langhendries JP Mostaert A. End-of-life decisions and practices for very preterm infants in the wallonia-brussels federation of Belgium. BMC Pediatr. (2018) 18:206. 10.1186/s12887-018-1168-x 29945564 PMC6020374

[42] Einarsdottir J. Emotional experts: parents' views on end-of-life decisions for preterm infants in Iceland. Med Anthropol Q. (2009) 23:34-50. 10.1111/j.1548-1387.2009.01036.x 19449711

[43] Janvier A Barrington K Farlow B. Communication with parents concerning withholding or withdrawing of life-sustaining interventions in neonatology. Semin Perinatol. (2014) 38:38-46. 10.1053/j.semperi.2013.07.007 24468568

[44] Bucher HU Klein SD Hendriks MJ Baumann-Hölzle R Berger TM Streuli JC. Correction to: decision-making at the limit of viability: differing perceptions and opinions between neonatal physicians and nurses. BMC Pediatr. (2018) 18:226. 10.1186/s12887-018-1204-x 29986696 PMC6038305

[45] Hendriks MJ Abraham A. End-of-life decision making for parents of extremely preterm infants. J Obstet Gynecol Neonatal Nurs. (2017) 46:727-36. 10.1016/j.jogn.2017.06.006 28751157

[46] Krick J Chabra S. On “end-of-life decision making for parents of extremely preterm infants”. J Obstet Gynecol Neonatal Nurs. (2018) 47:415-6. 10.1016/j.jogn.2018.01.007 29453954

